# Correlation Between Neck Circumference and Gestational Diabetes Mellitus and Associated Risk Factors During Pregnancy

**DOI:** 10.7759/cureus.2699

**Published:** 2018-05-28

**Authors:** Dr. KhushBakht, Saadon Mazhar, Amanullah Bhalli, Aqeela Rashid, Khurshid Khan, Uzma Jahanzaib

**Affiliations:** 1 Department of Internal Medicine, Fauji Foundation Hospital, Lahore; 2 Department of Diagnostic Radiology, Lahore General Hospital, Lahore; 3 Jinnah Allama Iqbal Institute of Diabetes and Endocrinology (jaide), Jinnah Hospital, Allama Iqbal Medical College, Lahore, PAK; 4 Department of Internal Medicine, Division of Endocrinology, Mayo Hospital Lahore

**Keywords:** neck circumference, gestational diabetes mellitus, correlation, anthropometric indices

## Abstract

Introduction

Due to normal physiological changes in various anthropometric indices during pregnancy, the routine measurements of body weight, height, waist circumference, and waist-to-hip ratio are deemed inappropriate in predicting obesity and risk of gestational diabetes mellitus in pregnancy. Neck circumference is a novel marker to determine the risk of gestational diabetes in pregnancy. We conducted this study to determine the correlation between neck circumference and gestational diabetes mellitus and its associated risk factors.

Methods

This was an observational, cross-sectional study conducted at Jinnah Allama Iqbal Institute of Diabetes and Endocrinology (JAIDE), Allama Iqbal Medical College/ Jinnah Hospital, Lahore from July 2017 to March 2018. Pregnant females at 16 weeks of gestation underwent measurement of weight, height, body mass index, neck, and waist circumference. At the 24^th^ gestational week, an oral glucose test was conducted and fasting lipid profile, serum albumin, and uric acid were measured. Pearson’s correlation was used to see any correlation between neck circumference and gestational diabetes and its associated risk factors.

Results

There were 90 subjects in the study with a mean age 30.8 ± 3.2 (range: 26 – 34) years. The waist and neck circumference at 16 weeks of gestation measured 104.2 ± 9.0 cm and 36.1 ± 2.8 cm, respectively. Age, weight, waist circumference, and body mass index were positively and significantly correlated with neck circumference (p-value < 0.05). After adjusting for age, the correlation between neck circumference and weight, waist circumference, and body mass index (BMI) was statistically significant with a p-value < 0.05. Analysis of the receiver operating curve revealed that the cut-off value of neck circumference for predicting gestational diabetes was 35.70 cm with a sensitivity of 51.4% and specificity of 81.2%.

Conclusion

Neck circumference at the 16^th^ gestational week is a reliable and valid tool to predict gestational diabetes mellitus at 24 weeks of gestation.

## Introduction

Gestational diabetes mellitus (GDM) is defined as any degree of serum glucose levels first intolerance with the onset and/or first recognition during pregnancy. The percentage of pregnant women with GDM is on the rise worldwide and ranges from 1% to 14% in different countries [1-2]. Furthermore, it is higher in Asian countries [3]. The reported prevalence of GDM among women in Pakistan has been reported to be 17.5% in one study [4]. To confirm that a pregnant woman has GDM, a standard oral glucose tolerance test (OGTT) must be administered at 24 - 28 weeks of gestation [5].

Gestational DM is another manifestation of insulin resistance and can be correlated confidently with metabolic syndrome [6]. It is well-accepted that diabetes and metabolic syndrome share similar risk factors. Generally, waist and hip circumferences and waist-to-hip ratio are used to support the diagnosis of metabolic syndrome [7]. However, none of these may give an accurate estimate during pregnancy since they are affected by many other factors and can change dramatically during pregnancy. The increase in the abdominal girth during pregnancy and changes in hip circumferences make it difficult for the clinician to accurately predict the risk of the development of gestational diabetes in pregnant women [8].

Neck circumference has been proposed to be an equal or better index than waist circumference and waist-to-hip ratio indicators for determining metabolic syndrome or its components [9]. It is easy to measure and has little inter-observer variability if measured in a pre-set manner. Several studies have reported it to be a useful tool in evaluating metabolic syndrome and associated risk factors, such as insulin resistance, central obesity, blood pressure, fasting glucose levels, and triglycerides [8, 10]. Since neck circumference has been shown to be strongly associated with insulin resistance, it has been postulated that it could be used as an indicator of diabetes mellitus. Its significance becomes more pertinent in pregnancy where results of other parameters become inconclusive and unreliable

Various studies have shown a positive correlation between neck circumference and associated gestational diabetes [11-12]. Since the concept is new, it has received scant attention in the recent medical literature from our country. Therefore, we conducted this study to determine the correlation between neck circumference and risk of gestational diabetes and associated risk factors in pregnant women.

## Materials and methods

This cross-sectional study was conducted at the Department of Gynecology and Obstetrics and Jinnah Allama Iqbal Institute of Diabetes and Endocrinology (JAIDE), Jinnah Hospital, Lahore from July 2017 to March 2018. The study was approved by the Ethical Review Board of Allama Iqbal Medical College/Jinnah Hospital, Lahore, Pakistan and was conducted using principles laid down in the Declaration of Helsinki 2001 [13]. A sample of 90 cases was calculated using the World Health Organization (WHO) sample size calculator with 95% confidence interval, 8% absolute precision, and anticipated population proportion to have gestational diabetes mellitus as 17.4% [11]. The patients were recruited following non-probability purposive sampling.

Gestational diabetes mellitus was defined on the basis of results of a two-hour, 75-gm oral glucose tolerance test (OGTT) conducted at 24 - 28 weeks of gestation as described by the American Diabetes Association. Patients who met at least two of these criteria were labeled as having gestational diabetes: fasting plasma glucose levels ≥ 5.1 mmol/L, a one-hour glucose level ≥ 10.0 mmol/L, and a two-hour glucose level ≥ 8.5 mmol/L. Neck circumference was measured in centimeters (cm) using a measuring tape at the level of the upper margin of the thyroid cartilage. A value > 35.1 cm was considered abnormal at 16 weeks of gestation.

The patients were recruited after taking an informed consent. Demographic information, such as maternal age, gestational age, gravidity, and parity, were collected. The following anthropometric parameters were measured at 16 weeks of gestation: a) neck circumference to the nearest 0.1 cm measured at the level of thyroid cartilage, b) body weight using a digital scale to the nearest 0.1 Kg, c) waist circumference to the nearest 0.1 cm using a measuring tape at the level of umbilicus, and d) blood pressure using manual sphygmomanometer in both arms followed by an average of the two readings. Body mass index was calculated by dividing the weight of the patient in kilograms by square of the height of the patients in meters (Kg/m^2^).

All patients also underwent an evaluation of a serum chemical profile at 24 weeks of gestation, including serum albumin, fasting lipid levels, and serum uric acid, conducted in collaboration with the Department of Pathology, Allama Iqbal Medical College, Lahore. The patients were required to observe an overnight fast for a minimum of 12 hours for analysis of various types of serum lipid levels. Enzymatic endpoint analysis was used for measurement of total cholesterol and triglycerides, and a precipitation analysis using phosphotungstic acid and magnesium was used for measuring high-density lipoprotein (HDL) after solidification of low-density lipoprotein (LDL) and very low-density lipoprotein (VLDL) in the ultracentrifuge. Patients with a previous history of gestational diabetes or diagnosed cases of diabetes mellitus were excluded from the study.

The collected information was analyzed using Statistical Package for Social Sciences (SPSS version 21.0, IBM Statistics Inc, Chicago, IL, USA). The mean and standard deviation were calculated for numerical variables, such as age, gestational age, body mass index (BMI), weight, blood glucose level, and waist and neck circumferences. Pearson’s correlation was used to find a relationship between neck circumference and gestational diabetes and its associated risk factors with p < 0.05 as statistically significant.

## Results

There were 90 participants in the study with a mean age of 30.8 ± 3.2 (range: 26 – 34) years. The mean gestational age at the time of delivery was 38.1 ± 3.1 (range: 37 – 40) weeks. The mean weight, height, and body mass index of the study population was 62.1 ± 18.2 Kg, 1.61 ± 0.03 m, and 23.8 ± 4.6 Kg/m^2^, respectively. The waist and neck circumference at 16 weeks of gestation measured 104.2 ± 9.0 cm and 36.1 ± 2.8 cm, respectively. The oral glucose tolerance test showed blood glucose level measuring 4.55 ± 0.7 mmol/L, 10.1 ± 1.9 mmol/L, and 9.0 ± 1.8 mmol/L at zero, one, and two hours, respectively (Table 1).

**Table 1:** Anthropometric and Clinical Profile of the Study Participants BMI: body mass index; SD: standard deviation

Variable	Mean ± SD (Range)	Range
Age (years)	30.8 ± 3.2	26 – 34
Weight (Kg)	62.1 ± 18.2	54 – 72
Height (m)	1.61 ± 0.03	1.5 – 1.8
BMI (Kg/m^2^)	23.8 ± 4.6	22.1 – 25.0
Waist Circumference (cm)	104.2 ± 9.0	96 – 112
Neck Circumference (cm)	36.1 ± 2.8	34.2 – 38.1
Blood Pressure (mmHg)		
Systolic	118.2 ± 10.1	110 – 128
Diastolic	78 ± 9.0	71 – 88
Fasting Blood Glucose (mmol/L)	4.81 ± 0.6	4.36 – 4.98
Oral Glucose Test (mmol/L)		
0 hour	4.55 ± 0.7	4.44 – 4.98
1 hour	10.1 ± 1.9	9.3 – 10.9
2 hours	9.0 ± 1.8	8.5 – 9.8
Serum Profile		
Triglycerides (mmol/L)	2.2 ± 0.9	1.9 – 2.7
Total Cholesterol (mmol/L)	5.4 ± 1.3	5.1 – 5.9
High Density Lipoprotein (mmol/L)	1.7 ± 0.4	1.4 – 2.0
Low Density Lipoprotein (mmol/L)	2.8 ± 0.8	2.5 – 3.0
Uric Acid (µmol/L)	350 ± 85	290 – 410
Serum Albumin (µmol/L)	36.1 ± 2.6	32.0 – 40.0
Gestational Age at Delivery (Weeks)	38.1 ± 3.1	37 – 40

The mean values of fasting lipid profile of the study participants that evaluated triglycerides, total cholesterol, HDL, and LDL came out to be 2.2 ± 0.9 mmol/L, 5.4 ± 1.3 mmol/L, 1.7 ± 0.4 mmol/L, and 2.8 ± 0.8 mmol/L, respectively. The mean levels of uric acid and serum albumin were 350 ± 85 µmol/L and 36.1 ± 2.6 µmol/L. respectively (Table 1).

We evaluated the correlation between neck circumference and various risk factors for gestational diabetes mellitus. Age, weight, waist circumference, and body mass index were positively and significantly correlated with neck circumference. The correlation coefficient (r) and p-values for age, weight, waist circumference, and BMI were r = 0.143, p = 0.031; r = 0.612, p < 0.05; r = 0.517, p < 0.05; and r = 0.501, p < 0.05, respectively (Table 2). The correlation with triglycerides, total cholesterol, fasting glucose level, and OGTT one-hour and two-hour glucose levels were not statistically significant with a p-value > 0.05 (Table 2). After adjusting for age, the correlation between neck circumference and weight, waist circumference, and BMI was statistically significant with a p-value < 0.05 (Table 3). Analysis of the receiver operating curve revealed that the cut-off value of the neck circumference for predicting gestational diabetes was 35.70 cm with a sensitivity of 0.514 and specificity of 0.812.

**Table 2:** Correlation Between Neck Circumference and Different Risk Factors of Gestational Diabetes Mellitus BMI: body mass index; hr: hour; OGTT: oral glucose tolerance test

Risk Factors	Correlation coefficient (r)	p-value
Age	0.143	0.031
Weight	0.612	0.00001
Waist Circumference	0.517	0.00001
BMI	0.501	0.00001
Triglycerides	0.102	0.061
Total Cholesterol	0.098	0.323
Fasting Glucose	0.071	0.438
OGTT 1-hr Glucose	0.078	0.401
OGTT 2-hr Glucose	0.069	0.517

**Table 3:** Correlation Between Neck Circumference and Different Risk Factors of Gestational Diabetes Mellitus After Adjustment for Age BMI: body mass index; hr: hour; OGTT: oral glucose tolerance test

Risk Factors	Correlation coefficient (r)	p-value
Weight	0.690	0.00001
Waist Circumference	0.545	0.00001
BMI	0.565	0.00001
Triglycerides	0.130	0.059
Total Cholesterol	0.101	0.218
Fasting Glucose	0.060	0.412
OGTT 1-hr Glucose	0.058	0.502
OGTT 2-hr Glucose	0.053	0.617

## Discussion

The primary objectives of the study to find a correlation between neck circumference and gestational diabetes mellitus and its associated risk factors were successfully met. The underlying main purpose of the study was to evaluate the validity of new indices, such as neck circumference, in predicting the risk of gestational diabetes mellitus in pregnancy that would ultimately help in the early detection and hence, the early management of gestational diabetes and would prevent complications associated with the disease. The results of our study showed that neck circumference measured at 16 weeks of gestation could be used as a predictor of gestational diabetes.

Neck circumference has been described as a marker of the distribution of upper torso fat and has been reported to correlate significantly with glycemic status, waist circumference, waist-to-hip ratio, and body mass index in fertile non-pregnant females [14-17]. Similarly, neck circumference has also been reported to correlate significantly with elevated levels of free fatty acids in the blood [18]. This is the reason it has taken its place as a reliable, valid, and strong predictor of visceral fat content and a marker of the state of insulin resistance in the body. Since the underlying pathophysiology of gestational diabetes mellitus has been shown to be insulin resistance [19], the neck circumference has been proposed to be a reliable predictor of gestational diabetes. Its importance becomes more crucial in pregnancy since anthropometric indices, such as waist circumference, body weight, and BMI, are affected significantly and become unreliable due to increased uterine volume in pregnancy. This is the reason that it has been suggested to explore other novel markers of body adipose tissue and insulin resistance that could predict the risk of gestational diabetes more accurately in pregnancy. However, since the concept is relatively new, not much literature is available on the subject [8].

He et al. reported conducting a similar study on the Han Chinese population and reported a neck circumference cut-off level of 35.15 cm to predict gestational diabetes in Chinese women with a sensitivity of 48.8% and specificity of 77.9% [11]. Similarly, Li et al. conducted a similar study in southern China that reported an even lower neck circumference cut-off level of 33.8 cm to predict gestational diabetes at 24 weeks of gestation with a sensitivity of 68.4% and specificity of 59.12% [12]. The current study found that the cut-off limit of neck circumference to predict gestational diabetes was 35.70 cm with a sensitivity of 51.4% and specificity of 81.2%. An earlier study by Yang et al. reported that a neck circumference of ≥39 cm for men and ≥35 cm for women was the best cut-off limit to determine subjects with metabolic syndrome. This study was conducted on the general population and did not address the cut-off limit in pregnancy. However, it conveyed the importance of neck circumference in predicting metabolic syndrome and reported the cut-off limits to be used in routine patients [20].

The results of the aforementioned studies that were exclusively conducted on pregnant females reported a lower cut-off level of neck circumference for predicting gestational diabetes than the one conducted on the general population. Its significance lies in the fact the sooner the clinicians find the reported cut-off limits of neck circumference in pregnant females, they should get an alert about the strong possibility of diagnosing gestational diabetes in those subjects and should closely monitor their glycemic status to diagnose and manage gestational diabetes well in time.

The study had some limitations as well. It was a single-centered study, and as we see different cut-off levels described in different studies above, we propose that larger multicenter studies are needed in our country to develop unanimous guidelines for cut-off levels to be used in our population. Secondly, we could not compare the results of our population with those pregnant female patients that did not develop gestational diabetes. Therefore, we recommend future case-control studies to compare the differences in various anthropometric indices and serum profiles between patients suffering from gestational diabetes and those who do not develop gestational diabetes. This would also give a head-on test to the neck circumference and will lead us to describe more its effectiveness in pregnancy as a strong predictor of gestational diabetes. However, the cut-off values reported in the current study are expected to increase awareness amongst clinicians about the new concept and would lead to the detection of gestational diabetes well in time. Lastly, the pregnant females can be asked to measure this index themselves and present to the doctor if they cross the cut-off limits for screening for gestational diabetes.

## Conclusions

Many indices of visceral adipose content become invalid in pregnancy due to increased body weight and abdominal girth. Neck circumference measured at 16 weeks of gestation is a reliable, valid, and easy to perform anthropometric index that can predict the development of gestational diabetes mellitus at 24 weeks of gestation with a strong sensitivity and specificity. Clinicians should add this measurement to their routine examination for early and timely detection and management of gestational diabetes mellitus.
